# Gastroesophageal reflux disease and the phantom of Barrett’s esophagus after most-often-used bariatric procedures: are future investigations necessary?

**DOI:** 10.1590/0102-67202025000041e1910

**Published:** 2025-11-10

**Authors:** Italo BRAGHETTO, Barbara CARREÑO, Ramón HERMOSILLA, Rafael ZANABRIA

**Affiliations:** 1 University of Chile, Faculty of Medicine, Hospital Clínico Dr. José J. Aguirre, Department of Surgery – Santiago, Chile.

**Keywords:** Bariatric Surgery, Esophagitis, Barrett Esophagus, Incidence, Cirurgia Bariátrica, Esofagite, Esôfago de Barrett, Incidência

## Abstract

**Background:**

Studies have investigated the incidence of gastroesophageal reflux disease (GERD) and Barrett’s esophagus (BE) after common bariatric surgeries. However, many of these studies have bias or limitations. Therefore, it is crucial to determine the true incidence of GERD in long-term follow-ups (FUs) post-surgery.

**Aims:**

The aim of this study was to review and summarize long-term data regarding the incidence of post-surgical GERD and BE after various bariatric procedures, discuss the characteristics of current information available, and establish the need for future studies to determine objective functional outcomes that have not yet been reported.

**Methods:**

A narrative review was conducted using multiple electronic databases, including the review of 15 meta-analyses and over 200 articles.

**Results:**

The quality of studies analyzing GERD and BE following bariatric surgery varies widely. Some papers provide detailed outcomes, while others offer limited information. The reported rate of *de novo* postoperative GERD development after sleeve gastrectomy varies from 4.06 to 74.7% (mean=33.8±19.1), and the incidence of BE ranges from 0.2 to 27% (mean=8.2±7.5). After Roux-en-Y gastric bypass (RYGB), similar variability is observed, with BE incidence ranging from 1.6 to 17.5% (mean=7.5±5.9). In the case of one-anastomosis gastric bypass (OAGB), scarce information is available and most reports are incomplete. The incidence of erosive esophagitis ranges from 15 to 70%, with BE incidence reported in only two papers (1–9.5%). For procedures such as single-anastomosis duodeno-ileal bypass with sleeve gastrectomy (SADI-S), fundoplication-sleeve, or sleeve bipartition, few specific data are available, with most reports limited to symptoms and lacking findings such as esophagitis, hiatal hernia, or BE.

**Conclusion:**

This revision provides evidence that SG may indeed lead to an increased risk of BE. Numerous studies suggest that RYGB protects against BE. Other bariatric procedures must be extensively evaluated. Relatively low quality of available literature on this topic was observed; therefore, well-controlled prospective studies with long-term FUs are necessary to fully understand the effect of bariatric surgery on BE.

## ARTICLE HIGHLIGHTS

It is well established that obese patients experience more severe esophagitis and Barrett’s esophagus (BE) than individuals of normal weight;

Bariatric procedures, designed to reduce excess weight, are expected to positively impact the prevention of gastroesophageal reflux disease and BE;

Current evidence suggests that the incidence of BE is higher following laparoscopic sleeve gastrectomy compared to the general population. This review provides compelling evidence that LSG may indeed lead to an increased risk of BE;

Numerous studies suggest that Roux-en-Y gastric bypass protects against BE.

## Summary

**CENTRAL MESSAGE:**

Literature on Barrett’s esophagus (BE) after bariatric procedure presents wide variation, and many studies have bias and limitations. In all reported cases, sleeve gastrectomy appears to promote gastroesophageal reflux disease and the potential development of BE, in contrast to Roux-en-Y gastric bypass (RYGB). Scarce and low-quality information is available regarding the other bariatric procedures.

**PERSPECTIVES:**

Current evidence suggests that the incidence of Barrett’s esophagus (BE) is higher following laparoscopic sleeve gastrectomy (LSG) compared to the general population. This review provides compelling evidence that LSG may indeed lead to an increased risk of BE. Numerous studies suggest that Roux-en-Y gastric bypass (RYGB) protects against BE. To fully understand the effect of bariatric surgery on BE, other bariatric procedures must be extensively assessed in well-controlled prospective studies with long-term follow-up (FU).

## VISUAL ABSTRACT

**Figure:**
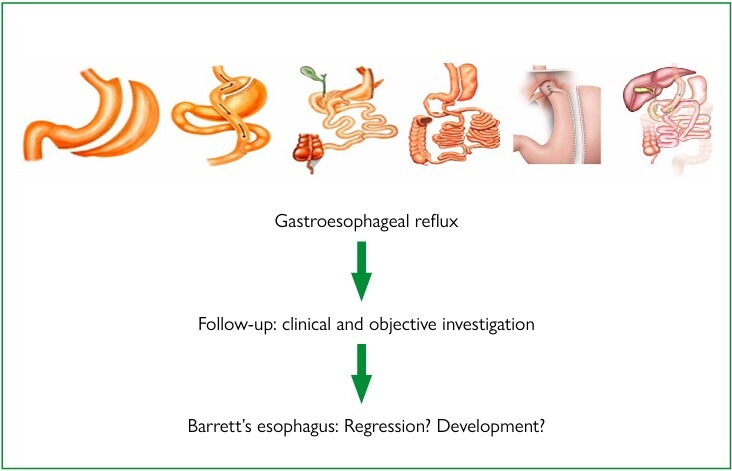

## RESUMO

**Racional:**

Estudos têm investigado a incidência de doença do refluxo gastroesofágico (DRGE) e esôfago de Barrett (EB) após cirurgias bariátricas habituais, mas muitos deles apresentam vieses ou limitações. Portanto, é crucial determinar a verdadeira incidência de DRGE em acompanhamentos de longo prazo após a cirurgia.

**Objetivos:**

Revisar e resumir dados de longo prazo sobre a incidência de DRGE e EB no pós-operatório tardio, após vários procedimentos bariátricos, discutir as características das informações atuais disponíveis e estabelecer a necessidade de estudos futuros para determinar desfechos funcionais objetivos que ainda não foram relatados.

**Métodos:**

Foi realizada uma revisão narrativa utilizando múltiplas bases de dados eletrônicas. Foram incluídos 15 meta-análises e mais de 200 artigos revisados.

**Resultados:**

A qualidade dos artigos que analisam DRGE e EB após cirurgia bariátrica varia amplamente, alguns fornecendo desfechos detalhados, enquanto outros oferecem informações limitadas. A porcentagem de ocorrência de DRGE pós-operatória *de novo* após gastrectomia vertical varia de 4,06 a 74,7% (média de 33,8±19,1%), e o esôfago de Barrett varia de 0,2 a 27% (média de 8,2±7,5%). Após bypass gástrico em Y-Roux também apresenta ampla variação, e a incidência de EB foi confirmada em taxas de 1,6 a 17,5% (média de 7,5±5,9). Após bypass gástrico com uma anastomose (OAGB), há poucas informações disponíveis e a maioria dos relatos é incompleta. A incidência de esofagite erosiva varia de 15 a 70%, com incidência de EB relatada em apenas dois artigos (1–9,5%). Poucos dados específicos estão disponíveis após procedimentos de *bypass* duodeno-ileal com anastomose única e gastrectomia vertical (SADI-S), fundoplicatura com *sleeve* ou bipartição com *sleeve*, relatando apenas sintomas, sem dados sobre esofagite, hérnia de hiato ou esôfago de Barrett.

**Conclusões:**

Esta revisão fornece evidências de que a cirurgia bariátrica, de fato, pode levar a um risco aumentado de EB. Numerosos estudos sugerem que o *bypass* gástrico em Y de Roux (RYGB) protege contra EB. Outros procedimentos bariátricos devem ser extensivamente avaliados. Foi observado uma qualidade relativamente baixa da literatura disponível sobre este tópico. Portanto, estudos prospectivos bem controlados com acompanhamento de longo prazo são necessários para compreender completamente o efeito da cirurgia bariátrica no EB.

## INTRODUCTION

 It is well established that obese patients experience more severe esophagitis and Barrett’s esophagus (BE) than individuals of normal weight^80^. Bariatric procedures, designed to reduce excess weight, are expected to positively impact the prevention of gastroesophageal reflux disease (GERD) and BE. Studies have investigated the incidence of GERD and BE after common bariatric surgeries, such as sleeve gastrectomy (SG) and Roux-en-Y gastric bypass (RYGB), but there is a need to evaluate other less common procedures such as one-anastomosis gastric bypass (OAGB), single-anastomosis duodenal ileostomy (SADI), fundoplication (FP)-sleeve (Nissen or Rosetti) (N-SG, R-SG), and sleeve with bipartition (SG-Bip)^1,15,31,33,34,36,120^. 

 Evidence suggests that the weight loss induced by these procedures could be a significant factor in reducing the risk of BE^54,99^. However, this conclusion is not entirely valid due to several biases and limitations in many studies. Conversely, the anatomical and mechanical changes resulting from these procedures could potentially contribute to the development of GERD and BE^7,23^. Therefore, it is crucial to determine the true incidence of GERD in long-term FUs post-surgery and identify which anatomical or pathophysiological factors must be investigated more deeply. The objectives are:

- To review and summarize long-term data (5–10 years) regarding the incidence of post-surgical GERD and BE after various bariatric procedures;
- To discuss the characteristics of current information available;
- To discuss whether development or regression of BE occurs following bariatric surgery;
- To establish the need for future studies to determine objective functional outcomes that have not yet been reported.

## METHODS

 A narrative review was conducted using multiple electronic databases (PubMed, MEDLINE, Google Scholar, and Scopus) with search terms such as "gastroesophageal reflux," "obesity," "Barrett’s esophagus," "bariatric surgery," "sleeve gastrectomy," "gastric bypass," and variations including "Roux-en-Y," "one anastomosis," "single anastomosis duodenal interposition," "gastric bipartition," and "Nissen-sleeve." We reviewed 15 meta-analyses and over 200 articles, but only those with long-term FU data and written in English were included. Studies were selected based on pre- or postoperative occurrence of BE in patients undergoing bariatric surgery, excluding those with unavailable full texts, early postoperative data, or written in other languages. 

## RESULTS

 Numerous papers have examined GERD and BE following bariatric surgery. The quality of these studies varies widely, with some providing detailed clinical and functional outcomes, while others offer limited information. Symptoms of GERD show a broad range, with SG alone or combined with other procedures generally associated with more frequent symptoms. The same trend is observed in the use of proton pump inhibitors (PPIs) and the presence of varying grades of esophagitis post-surgery. BE is most commonly observed after SG, also reported following RYGB, especially when the surgical technique was not properly executed. Data on other bariatric procedures are scarce, and cancer development post-surgery is rare and difficult to assess^2,10,38,42,60,72,76,77,112^ (Table 1). 

**Table 1:** Summary of GERD consequences after different bariatric procedures^2,10,38,42,60,72,76,77,112^.

		SG (%)	RYGB (%)	OAGB (%)	SADI (%)	N-SG (%)	SG-Bip (%)
GERD symptoms		15.2–74.7	2–22	1.6–52.1	6.3–7.5	36.6	1–22
PPI use		13–61	7.4–64	19.3–27.1	No data	No data	2–7
Esophagitis (%)		33.1–44.4	5.8–17.6	15–29.1	No data	No data	
	A	19–92	5.1–57.1				10
	B	19.4–32.7	6.2				16
	C	11.8–18					4
	D	9.1					
Barrett’s esophagus		2.2–27.2	0–17.6	1–9.5	No data	No data	No data
Hiatal hernia		1.2–45	20–53.2	29.2	No data	No data	No data
Esophageal carcinoma		0.05% of cases reported	0.003%	5 cases reported	No data	No data	No data

GERD: gastroesophageal reflux disease; PPIs: proton pump inhibitors; SG: sleeve gastrectomy; RYGB: Roux-en-Y gastric bypass; OAGB: one-anastomosis gastric bypass; SADI: single-anastomosis duodenal ileostomy; N-SG: Nissen fundoplication-sleeve, and SG-Bip: sleeve with bipartition.

Table 2 presents data reported over the past decade following long-term FU after SG. Most authors agree on the high percentage of symptoms (affecting nearly 80% of patients), the presence of esophagitis, and the use of PPIs. The rate of de novo postoperative GERD development varies from 4.06 to 74.7% (mean=33.8±19.1) and that of BE ranges from 0.2 to 27% (mean: 8.2±7.5). Notably, only seven out of 32 papers (21.9%) reported more than 10% incidence of BE. In contrast, several studies reported hiatal hernia (HH) incidence rates exceeding 20%. 

**Table 2:** Results of the reported incidence of GERD and Barrett’s esophagus after SG.

Author	Year	Follow-up (months)	PPI use (%)	Esophagitis A-B-C (%)	Barrett’s esophagus (%)	Hiatal hernia (%)	Cancer (%)	GERD symptoms (%)	Incompetent LES (%)
Catheline et al.^31^	2013	60	33.3						
Qumseya et al.^93^	2021	60	7.4		11.6				
Kular et al.^61^	2014	60	21					21	
Rawlins et al.^94^	2012	60						11	
Sebastianelli et al.^104^	2019	78±15	52	41	18.8			76	
Felsenreich et al.^43^	2022	120	70	44	13	50		55	
Soricelli et al.^110^	2018	66	63.9	60.1	13.1			70.2	
				A=22.2					
				B=19.4					
				C=18.5					
Csendes et al.^36^	2019	126	120	58.5	4	18.9			
Braghetto et al.^24,26^	2016	36		15.5		1.2			
	2019	60				4.8			
Dimbezel et al.^41^	2020			35.6				27.1	
Genco et al.^50,51^	2021	58	68.1	74.7	17.2		3 cases	58.9	
				A=46.3					
				B=32.7					
				C=11.8					
				D=9.1					
Alexandrou et al.^9^	2015	60						16	
Angrisani et al.^14^	2016	60						8–15	
Ferrer et al.^48^	2022	60		31.4	0.9	30.5		76	
Arman et al.^17^	2016	140.7	11.1					21.4	
Al Sabah et al.^7^	2021	60		15.2	2.2	4.4			
Tai et al.^115^	2012	12		66.7		27.3		47	
				A=36.4					
				B=24.2					
				C=6.1					
Braghetto et al.^24^	2016	60		15.5	4.8	5.7		15.5	85.1
									77.5
Yeung et al.^125^		3–132							
Benvenga et al.^19^	2020	66–85.2		19.2	1.3	23.1			
Bevilacqua et al.^20^	2020			4.06	0.84		0.08		
Salminen et al.^99^	2022	120	64	31	4	63			
Lallemand et al.^62^	2020	60		27.1	8.5	42.4		50.8	
Migaczewski et al.^78^	2021	60		30	27			56.7	
Swei et al.^113^	2023			14	6			54	
Kermansaravi et al.^59^	2023	60		29.5	5.7				
				A=15.2					
				B=11.4					
				C=2.9					
Moulla et al.^80^	2023			30					
Thereaux et al.^117^	2017	48	21						
Navarini et al.^83^	2020	12		51.4				60	40
Matar et al.^77^	2020	60	73.6	37.9	1.1				16.9
Leslie et al.^67^	2021	124	4.7	12.1	0.7			60.2	39.3
Mean±SD		68.7±32.8	44.3±34	33.8±19.1	8.2±7.5	26.4±20.4		44.9±22.6	51.8±28.6

PPIs: proton pump inhibitors; GERD: gastroesophageal reflux disease; SD: standard deviation; LES: lower esophageal sphincter.

 Genco et al.^51^ recently reported three cases of adenocarcinoma after SG. Although the rate and probability of progression from BE to esophageal adenocarcinoma (EAC) are not well defined, the increasing popularity and execution of SG may lead to a rise in BE incidence. Clinical and endoscopic FUs are essential for prevention, early diagnosis, and epidemiologic data collection. Gastric reflux worsened more often after SG (31.8%) than after RYGB (6.3%)^8,9,14,17,19,20,24,26,32,37,41,43,44,48,50,51,59,61,62,67,77,78,80,83,84,88,93,94,104,110,113,115,117,125,128^. 

 Papers reporting data on GERD and BE after RYGB also show wide variation in incidence (Table 3), with some focused solely on postoperative symptoms, while others included objective results from endoscopic studies^12,80,100,112^. These papers reported incidences of various grades of esophagitis, ranging from 0.4 to 26.7%, with most being grade A, and some authors noting grade C or D esophagitis. BE incidence was confirmed at rates from 1.6 to 17.5% (mean: 7.5±5.9). Adil et al.^3^, in their meta-analysis, reported the percentage of BE and response to RYGB but warned about the quality of reports (low or intermediate). Only two papers described BE rates over 10%, likely due to technical errors^50^. Conversely, Csendes et al. demonstrated a very low rate of symptoms, erosive esophagitis, BE, and HH after 10 years of FU, probably due to a very small gastric pouch (4.46 cm)^3,21-23,25,35,45,50,53,77,99,100,117,123^. Data regarding manometry and pH monitoring are scarce. 

**Table 3:** Results of the reported incidence of GERD and Barrett’s esophagus after RYGB.

Author	Year	Follow-up (months)	PPI use (%)	Esophagitis A-B-C-D (%)	Barrett’s esophagus (%)	Hiatal hernia (%)	Cancer (%)	GERD symptoms (%)
Borbély et al.^22^	2018	45.6	57.4	51.1	14.9	53.2		
				A=23.4				
				B=17				
				C=4.3				
				D=6.4				
Braghetto et al.^24^	2022	60		28.9	5.7	40		
				A=18.9				
				B=5				
				C=2.5				
				D=2.5				
Santonicola et al.^100^	2022	60–130						24.4
Wölnerhanssen et al.^123^	2023	84±12	19.8	27	1.6	18.6		26.7
Felsenreich et al.^44^	2020		14.6	12.9	12.9	0		24.4
Salminen et al.^99^	2022	120	28	7	5			
Boerlage et al.^21^	2020	7		2				
Huang et al.^53^	2003	49		0.4				
Braghetto et al.^24^	2016	60			3			
Genco et al.^50^	2021	120	24.4	22	17.5			
Navarini et al.^83^	2020			15		17		10
Matar et al.^77^	2020	60	62.5	17.6	5.1			16.6
Csendes et al.^35^	2022	120		5.4	3.2	2.2		6.5
Andrew et al.^12^	2018							4.7
Leslie et al.^67^	2021					1.1		35.3
Mean±SD		72.6±37.9	34.5±20.3	17.2±14.7	7.5±5.9	18.9±20.7		18.6±10.2

PPIs: proton pump inhibitors; GERD: gastroesophageal reflux disease; SD: standard deviation.

 The literature on GERD data after OAGB is scarce compared to the extensive reports after SG or RYGB. Most reports are incomplete, confusing, and of poor quality due to incomplete data and inconclusive findings. Reflux symptoms after OAGB vary widely, ranging from 0 to 55%, and PPI use is as high as 51.1%. The incidence of erosive esophagitis ranges from 15 to 70%, with BE incidence reported in only two papers (1–9.5%), indicating unreliable data (Table 4)^8,16,42,46,47,50,58,60,63,68,69,75,82,89,90,92,95,98,103,105,106,108,109,114,117,121^. 

**Table 4:** Results of the reported incidence of GERD and Barrett’s esophagus after OAGB.

Author	Year	Follow-up (months)	PPI use (%)	Esophagitis A-B-C (%)	Barrett’s esophagus (%)	Hiatal hernia (%)	Cancer (%)	GERD symptoms (%)
Landreneau et al.^63^	2019	36						18.8
Antonopulos et al.^16^	2020	34						60.9
Sargsyan et al.^103^	2024							47.8
Felsenreich et al.^43,47^	2022	48		28.3	9.5	0		28.3
	2023	60		33.3	11.1	0		
Esparham et al.^42^	2023			15	1			6
Kermansaravi et al.^60^	2020			19.3		29.2		
Nehmeh et al.^84^	2021			11.6	4.6			
Davarpanah et al.^38^	2023							0–55
Genco et al.^50^	2021		27.1					52.1
Soprani et al.^109^	2020	60		74				
Szymański et al.^114^	2022	24		48	8			6
Sohrabi et al.^108^	2020	60		10.6		6.6		
Slagter et al.^106^	2021	36		3	2			57
Shenouda et al.^105^	2018	6		45				55
Salama and Hassan^98^	2017	12		4				
Robert et al.^95^	2019	24		10.1	1.7			7.7
Plamper et al.^90^	2023	25		8.3				
Pizza et al.^89^	2020	24	5.7	2.9				5
Mustafa et al.^82^	2020	24		8.5	0.5			13.5
Mahdy et al.^75^	2023	12		11				10.8
Katayama et al.^58^	2021	6		70				
Mean±SD		27.36±17.01	16.40±15.13	23.70±22.79	4.80±4.18	7.90±15.98		28.38±22.59

PPIs: proton pump inhibitors; GERD: gastroesophageal reflux disease; SD: standard deviation.

 Deffain et al.^39^ conducted a retrospective study on 179 patients regarding GERD after single-anastomosis duodenoileal bypass with sleeve gastrectomy (SADI-S). Four years of FU showed that 33% had GERD and 25% used PPIs postoperatively to control their symptoms, though the method of reporting these findings was not described. Yashkov et al.^124^ reported GERD symptoms in 7.5% of patients, Surve et al.^111^ in 12.5%, and Admella et al.^4^, in a prospective study of 246 patients, noted a progressive increase in GERD after SADI-S, rising from 18.8 to 30.2% over 3 years. These papers did not report on esophagitis, HH, or BE postoperatively^111,124^ (Table 5). 

**Table 5:** Reported results of GERD after SADI’s procedures.

Author	Year	Follow-up (months)	PPI use (%)	GERD symptoms (%)	Barrett’s esophagus (%)	Hiatal hernia (%)
Deffain et al.^39^	2024	48	25	33	NR	NR
Yashkov et al.^124^	2021	60		7.5	NR	NR
Surve et al.^111^	2020	9		12.5	NR	NR
Admella et al.^4^	2023	36		30.2	NR	NR
Mean±SD		38.25±21.82		20.8±12.67		

PPIs: proton pump inhibitors; GERD: gastroesophageal reflux disease; SD: standard deviation; NR: not reported.

 Few specific data are available on outcomes after SG plus FP or HH repair (Table 6). These papers focus mainly on the presence or remission of GERD symptoms, with few reporting on esophagitis, BE, or HH post-surgery. The overall quality of evidence is low due to the observational study design, heterogeneity, lack of blinded outcome assessment, short FU periods, and evidence of publication bias. However, the combination of antireflux maneuvers with SG appears to improve preoperative symptoms. The reported rate of GERD symptoms varies depending on the FU period, with BE mentioned in only one paper. Regarding the incidence of BE post N-SG or R-SG, the rate of de novo BE after 2 years is nil, though one paper reported one case of adenocarcinoma after 5 years of FU^5,6,11,13,18,27,29,30,34,65,71,73,79,81,86,87,118,119^. 

**Table 6:** Summary of reported data regarding GERD and Barrett’s esophagus after sleeve gastrectomy combined with antireflux procedures.

Surgery	Years	Follow-up (months)	GERD postop (%)	*de novo* GERD (%)	Esophagitis (%)	Hiatal hernia (%)	Barrett’s esophagus (%)
SG+HH repair^18,30,73,81^	2013 2024	12–48	20.4±17.5	12–30.6	30.2–55	11–55	1.1
SG+FP^29,65,79,88,118^ Nissen SG^11,27^ Rossetti SG^86,87,119^ Toupet SG^118^ DOR SG^70^ Posterior^18^ Dor-SG-Bipart^126^	2017 2022	22–60	5±8.1 4.8 1.6 6% 4.1±5.8 13.6% 15%	11–13.6	2–20	NR	0 0

GERD: gastroesophageal reflux disease; SG: sleeve gastrectomy; HH: hiatal hernia; FP: fundoplication; Bipart: bipartition; DOR: Dor fundoplication.

 For GERD post-SG with Bipart, 32 papers were reviewed. Only four included data on GERD after the procedure, with one paper reporting a 12% incidence of GERD symptoms. There was no mention of esophagitis, HH, or BE^102,105^. 

 Data regarding esophagitis or BE regression or progression after other antireflux procedures such as Hill gastropexy, use of the ligament of Teres, and sphincter augmentation with the Linx device are not available. 

## DISCUSSION

 This study focused on BE development after bariatric procedures, as GERD symptoms, esophagitis, and other GERD-related issues have been sufficiently analyzed previously. A key observation is the lack of updated information on less common bariatric surgeries regarding BE. There is significant variability in the focus of published papers: some discuss results in terms of symptoms but do not detail the specific characteristics of patient evaluations. Most current studies are conducted on SG, followed by RYGB. Data on OAGB are scarce because it is relatively newer, and no long-term studies have been reported. The situation is worse for SADI-S or SG combined with antireflux procedures, as the literature is insufficient to analyze the rate of BE development. 

 It has been shown that BE development is associated with obesity, making it reasonable to suggest that bariatric surgery would protect against its development. The reduced risk of BE diagnosis in bariatric surgery patients could potentially be attributed to improvements in metabolic factors such as weight and body mass index (BMI). While weight loss is likely the primary driver of BE risk reduction, other mechanisms may also be at play, including mechanical, anatomic, and hormonal changes. The current data on de novo BE may not be very accurate due to many factors, such as missing anatomic and pathophysiologic considerations that play a role in BE development or improvement after different bariatric procedures, and mainly due to unsuitable FU. 

 Reflux may be assessed both subjectively (clinical symptoms) and objectively (upper endoscopy, monitoring, esophageal manometry, and histologic findings in biopsy). However, these parameters are missing in most studies, making the results of this meta-analysis heterogeneous. Few papers provide complete postoperative evaluations. Additionally, many studies have short and incomplete FUs, which are insufficient for the correct diagnosis of BE. BE results from chronic acid and bile reflux over more than 5–10 years of reflux disease evolution. 

 Despite the lack of periodic endoscopic or histological evaluations throughout the FU, we must consider the current reported data on the postoperative incidence of BE after bariatric procedures. 

 The mechanisms involved in the development of GERD and BE after SG are well described in the literature, supporting the high incidence of BE, as confirmed in this review. This is very similar to the International Federation for the Surgery of Obesity and Metabolic Disorders (IFSO) report in 2018 which is as high as 4.6–6% within 5 years after surgery^24,26,37,41,43,44,49,62,78,104,110,113,115,125^. This finding is concerning, as the risk of BE in the general population is only 1–2%. If the estimates from current and other studies are correct, performing LSG could create a true "at-risk" population, with up to a six-fold higher risk of BE development^106^. Conversely, two papers reported no significant difference in BE risk between SG and RYGB cohorts (36% versus 5%; OR (odds ratio)=5.73; 95%CI (confidence interval) 5–5.11) and even suggested that bariatric surgery might protect against BE^5,6^. However, this study had many biases and limitations^23^. 

 In two European studies, a 5-year FU showed that 17–19% of patients who underwent SG developed de novo BE^50,61^. The Swiss Multicenter Bypass or Sleeve Study (SM-BOSS) randomized clinical trial indicated that gastric reflux worsened more often after SG (31.8%) than after RYGB (6.3%)^88^. BE after RYGB has been established as the procedure of choice for patients with GERD due to its protective action against disease progression. Interestingly, some recent studies have also found that RYGB may be associated with BE, dysplasia, and EAC development at variable periods following surgery^49,108,109^. Although gastric refluxate after RYGB is less acidic, it might still prove harmful to the esophagus, warranting further study^25,35^. 

 Many authors have demonstrated BE regression after RYGB, a parameter not included in Table 3. According to Gorodner et al.^52^, laparoscopic RYGB (LRYGB) is a suitable treatment for obese patients with BE, demonstrated by a 36% regression rate of this premalignant disease. Although BE persisted in the remaining patients, no progression to dysplasia was observed. More patients and longer FUs are needed for definitive conclusions. In a review including 28 articles, no cases of BE progression were reported. Postoperative BE regression rates varied from 36 to 62%. Another meta-analysis showed GERD resolution in 74.2% of the LRYGB group versus 28.0% in the LSG group. Based on our results, we recommend LRYGB for patients with severe reflux symptoms^20,52,70^. 

 Regarding BE after OAGB, the rates of esophagitis and BE were 15 and 1–6%, respectively. Many studies did not specify how they diagnosed GERD, with some relying solely on clinical symptoms or upper endoscopy, and fewer evaluating manometry or pH monitoring. These few papers demonstrated acidic reflux in few patients, with bile reflux noted during endoscopy. Transformation from reflux esophagitis to Barrett’s metaplasia, likely caused by bile reflux after OAGB, has been reported^42,64^. 

 Although OAGB can theoretically induce chronic biliary reflux, the incidence of biliary reflux and cancer risk has not been prospectively evaluated. Gastroesophageal reflux is a clear cause of BE metaplasia and adenocarcinoma of the esophagogastric junction (AEG). Although rare, five gastric cancers and two cases of AEG have been published. Clarification of this issue is urgently needed^28,38,40,46,47,57,60,66,92,96,97,107,116,126,127^. Unfortunately, our review confirmed the lack of long-term studies on de novo BE appearance or regression. 

 Regarding the SADI-S procedures reported by Deffain et al.^39^, Admella et al.^4^, Yashkov et al.^124^, and Surve et al.^111^, involving more than 500 patients, only GERD symptoms were reported. None of these studies provided objective mention of esophagitis, HH, or BE, nor did they include manometry or pH monitoring. 

 Studies have shown that SG with concomitant FP and HH repair is effective for reflux resolution and should be considered as an alternative to conventional SG in patients with obesity and HH and/or GERD. High-resolution impedance manometry (HRiM) showed increased lower esophageal sphincter (LES) function, and multichannel intraluminal impedancepH (MII-pH) showed excellent control of both acid exposure and reflux events. 

 For patients unwilling or unable to undergo RYGB, SG may offer a safe and effective option to avoid BE development, even for those with pre-existing BE. However, NSG remains under evaluation. Aiolfi et al.^6^ estimated an 11% postoperative GERD prevalence, and Castagneto-Gissey et al.^30^ reported a 5% postoperative GERD rate. Further studies with extended FU and direct comparisons to conventional SG are warranted^40,65^. 

 However, definitive and complete evaluations after long-term FU are missing. 

 Santoro et al.^102^ and Zhao et al.^126^ presented SG with transit bipartition (TB) as a surgical alternative effective in weight loss and GERD remission. SG+TB is potent for treating metabolic syndrome and obesity, addressing both obesity and GERD without mechanical restriction and significant malabsorption^101,102,126^. An experimental study showed that the TB procedure might protect the distal esophagus from histological changes associated with esophagitis, although clinical studies are needed to confirm these anti-reflux effects^122^. 

 Regarding cancer appearance after bariatric surgery, the incidence of esophageal cancer has not been well determined in large longitudinal cohort studies. Current evidence is limited, showing no difference in incidence between bariatric surgery patients and non-surgical obese patients^20,57^. Lazzati et al.^66^ reported a significant reduction in esophageal and gastric cancer incidence following bariatric surgery in a nationwide cohort. However, their multivariate analysis showed no significant difference between SG and RYGB regarding cancer incidence (hazard ratio [HR]=1.6, 95%CI 0.9–2.7, p=0.09 for SG versus RYGB). Conversely, other authors suggest that SG may increase the risk of BE development (0.02%) due to mechanisms that increase clinical GERD and progression to BE and potentially adenocarcinoma. Conversely, RYGB appears to be protective against disease progression to neoplastic BE during endoscopic surveillance (0.003%). Although acid/bile reflux decreases after RYGB, disease progression has still been observed, warranting continued endoscopic surveillance. The effect of RYGB on the development or progression of BE or EAC remains unclear^8,16,55,56,63,74,85,91,98^. 

 A significant limitation of this article is the consequence of the relatively low quality of the available literature on this topic. This is due to the low rate of BE detection before bariatric surgery and the lack of routine endoscopic screening before and after bariatric surgery in most studies. Additionally, there is often a lack of adherence to protocols such as the Prague classification or Seattle protocols for biopsies, small patient numbers, considerable heterogeneity, non-prospective studies, and short-term FUs. These factors introduce biases that could confound the results. Furthermore, inter-observer variation in the diagnosis of BE across the included studies may lead to observer bias. The postoperative use of PPIs, although clearly mentioned in many reports, could also potentially confound the results of the various meta-analyses. 

 The strengths of this investigation include an extensive data search encompassing reviews, meta-analyses, cohort series, and prospective studies. This critical analysis of the obtained data determines the current results, identifies deficiencies in the consulted articles, and highlights the need for prospective studies with comprehensive objective evaluations of patients. The IFSO supports further high-quality studies in this field, mainly prospective and/or population-based studies, to elucidate the exact magnitude of the issue and provide further guidance to the community as necessary. Researchers should particularly focus on identifying potentially confounding factors when assessing the impact of certain procedures on the development and/or progression of BE^49^. 

## CONCLUSIONS

 Current evidence suggests that the incidence of BE is higher following LSG compared to the general population. This review provides compelling evidence that LSG may indeed lead to an increased risk of BE. Numerous studies suggest that RYGB protects against BE. To fully understand the effect of bariatric surgery on BE, other bariatric procedures must be extensively evaluated with well-controlled prospective studies with long-term FU. 

## Data Availability

The Informations regarding the investigation, methodology and data analysis of the article are archived under the responsibility of the authors.
